# Advanced vaccinology education: Landscaping its growth and global footprint

**DOI:** 10.1016/j.vaccine.2020.05.038

**Published:** 2020-06-19

**Authors:** Edwin J. Asturias, Philippe Duclos, Noni E. MacDonald, Hanna Nohynek, Paul-Henri Lambert, the Global Vaccinology Training Collaborative

**Affiliations:** a University of Colorado School of Medicine, 13199 East Montview Blvd., S-310 Aurora, CO, USA; b The Jules Amer Chair in Community Pediatrics, Children’s Hospital Colorado, 13123 E 16th Ave, Aurora, CO 80045, USA; c University of Geneva, Centre for Vaccinologie, 1 rue Michel Servet, 1211 Geneve 4, Switzerland; d Department of Pediatrics, Dalhousie University, IWK Health Centre, 5850/5980 University Ave, Halifax, Nova Scotia B3K 6R8, Canada; e National Institute for Health and Welfare, Department of Vaccines and Immune Protection, 166 Mannerheimintie, P.O. Box 30, FI-00271 Helsinki, Finland

**Keywords:** Vaccinology, Education, Vaccines, Training, Global, Survey

## Abstract

In preparation for the first Global Vaccinology Training workshop in 2018, a survey of 27 advanced vaccinology courses was conducted to provide a landscape of the vaccinology education around the world. Advanced vaccinology courses have expanded dramatically over the last 20 years, with courses located in almost all regions, but with underrepresentation amongst the Eastern part of the European region, the Eastern Mediterranean and the Western Pacific regions. Most courses are of short duration (<2 weeks), have a global or regional reach, and attract a diverse range of participants from high, middle and low-income countries with representation from public health, academia, industry and less often regulators. Lack of sustainable funding and time commitments of faculty and coordinators is a constraint for most vaccinology courses and needs to be addressed. Continuation and extension of training in vaccinology worldwide will be necessary as increasing number of new and more complex vaccines are introduced, vaccine safety concerns and rumors continue their trend, and reemergence of some vaccine-preventable diseases will require a competent workforce to advance and deploy immunizations to larger populations.

## 1 Introduction

For over a century, immunization has been one of the best recognized and most effective and equitable public health interventions to decrease morbidity and mortality from infectious diseases around the world. Optimizing the development, implementation and effectiveness and safety of immunization requires a well-trained work force. The Global Vaccine Action Plan (GVAP) — endorsed by the 194 Member States of the World Health Assembly in May 2012 — outlines the importance of ensuring that immunization programs are adequately staffed with personnel who are well trained [1], [2]. Education in vaccinology is a cornerstone strategy to strengthen not only the development of vaccines, but also their deployment, assessment of safety, effectiveness and uptake evaluation [3].

While Jonas Salk was not the first to coin the term vaccinology, he suggested a more comprehensive definition than earlier efforts, explaining it as “the study and application of the requirements for effective immunization… *a* body of knowledge *that* would include an understanding of the fundamental properties of the immune system and of specific immunogens…the diseases’ etiological agents, pathogenic mechanisms, and their epidemiology… Applied vaccinology would involve the application of basic knowledge and practical solutions to the development of effective vaccination programs suitable for particular population groups” [4], [5].

The need for advanced education in vaccinology as one means to strengthen the immunization workforce around the world was recognized by a group of vaccinology experts in 1999, and materialized as the first Advanced Vaccinology Course (ADVAC) in 2000 [6]. Since then, academic centers, foundations dedicated to the advancement of vaccines, as well as policy makers have organized advanced vaccinology courses in different regions of the world with the aim of enhancing the education in vaccinology of researchers, clinicians, educators, regulators, manufacturers and public health officials.

In late 2018, ADVAC convened a Global Vaccinology Training workshop, to bring together leaders from vaccinology courses around the world to review the courses’ aims, structure, format and types of participants in order to identify education gaps and future needs and also to discuss potential collaboration [7]. In preparation for the workshop, we designed and conducted a pre-workshop survey of vaccinology courses identified and invited to join at the workshop, in order to provide a description of their organization, funding, audience, educational methods, trials and tribulations. This paper summarizes the findings of that landscape analysis.

## 2 Methods

This landscape analysis was designed as a cross-sectional survey of post-graduate vaccinology courses in existence around the world. Via internet search, cross-referencing and expert inquiry we identified 39 vaccinology courses in 2018. University-based undergraduate (i.e. preservice) and graduate courses being taught as part of a degree (with few exceptions), courses that only focused on one issue of vaccinology, and periodic courses or symposia on vaccinology were excluded (n = 12). In the selection, preference was given to currently available courses, geographic diversity, and low- and middle-income country representation. Thus, the leaders of 27 selected courses were invited to participate in the workshop and complete a standardized questionnaire. This 27-question survey comprised information on the course organization, coordination, collaborating partners and funding sources, course participant selection process including background, geographic and their economic status; course objectives and curricula, format of training, evaluation process, and post-course activities and communication [7]. The survey instrument was sent by e-mail to the identified course directors, allowing a 3-month period for response. Email reminders and follow-up phone call contacts were made to ensure responses before the workshop and clarify answers as necessary.

Data was subsequently entered in Microsoft® Excel 2018 (version 16.16.4, Redmond, WA, USA) and analyzed using Stata® 14.2 (StataCorp, College Station, TX, USA). We summarized data descriptively using frequencies for categorical variables and measures of central tendency for continuous variables.

The survey was considered non-human subjects research and quality improvement, and thus was not submitted for Institutional Ethics Board review.

## 3 Results

Of the 27 courses solicited, 26 (96.3%) responded to the email invite agreeing to participate in the workshop and completed the questionnaire (data summarized in Table 1). Of the 26 advanced vaccinology courses, 14 (53.8%) reported targeting global (with some language restrictions), 8 (30.8%) regional, and 4 (15.4%) national audiences. Most courses were offered annually (21, (80.8%)), while the remaining were provided every other year. Given that most of vaccinology training is aimed at postgraduate participants, 11 (42.3%) are delivered in one-week and 6 (23.1%) in a two-week period. The other 7 (26.9%) that reported their duration were first created as a month-long presential training (Institute Pasteur, France) or are on-line training courses or Master’s degree programs.

**Table 1:** Characteristics of selected Advanced Vaccinology Courses around the world by 2018.

Name	Country	WHO Region	Year Established	Reach	Language	Duration (weeks)	Total Participants Trained	Average participants per year	Distribution				
									Academics	Public Health	Industry	Regulatory	Other
India Advanced Vaccinology Course	India	SEAR	2010	N	E	2	348	44	70%	9%	15%	7%	0%
Vaccinology in Africa Master's Course	Africa	AFR	2013	R	E	1	142	36	83%	2%	15%	0%	0%
Oxford Vaccinology Course	UK	EUR	2009	G	E	1	373	37	60%	0%	20%	20%	0%
TropEd Advance Vaccinology Course	Germany	EUR	2001	G	E	2	400	24	90%	0%	10%	0%	0%
Master in Vaccinology and Pharma CD	Italy	EUR	2009	G	E	77	72	14	13%	33%	31%	10%	13%
Epidemiological evaluation of vaccines	UK	EUR	2013	G	E	2	164	33	15%	60%	10%	5%	0%
Multidisciplinary Vaccinology	Canada	AMR	2008	G	E	42	95	10	NR	NR	NR	NR	NR
Vaccinology for Immunization Managers	Argentina	AMR	2011	R	S	1	243	35	15%	80%	0%	5%	0%
Regional Vaccinology Course Africa	AFRO	AFR	2007	R	EC	1	327	41	26%	72%	0%	0%	2%
Vaccinology Summer School	Netherlands	EUR	2018	G	E	1	0	0	NR	NR	NR	NR	NR
Masters of Advanced Studies Vaccinology	Switzerland	EUR	2016	G	E	80	10	5	NR	NR	NR	NR	NR
Clinical Vaccinology Course	USA	AMR	2006	N	E	0.5	2300	192	5%	90%	0%	0%	5%
International Vaccinology Course	Korea	WPR	2000	G	E	1	1207	67	30%	40%	20%	10%	0%
IP International Vaccinology Course	France	EUR	2008	G	E	4	274	27	55%	35%	5%	5%	0%
MOOC Vaccinology	France	EUR	2015	G	E	6	5500	2750	30%	30%	30%	10%	0%
Vaccinology Course	India	SEAR	2011	N	E	1	275	69	10%	40%	40%	10%	0%
ECAVI Vaccinology Course	Uganda	AFR	2016	R	E	1	500	80	30%	60%	0%	10%	0%
International Course on Vaccinology	Senegal	AFR	2012	R	F	NR	145	24	10%	10%	80%	0%	0%
Latin American Online Vaccinology	Mexico	AMR	2009	R	S	5	4300	478	10%	60%	10%	20%	0%
Chinese Vaccinology Course	China	WPR	2018	N	EC	1	70	70	20%	53%	20%	7%	0%
French International Vaccinology	France	EUR	2006	G	F	2	200	18	20%	0%	80%	0%	0%
Summer Course on Vaccinology	Belgium	EUR	2009	R	E	1	366	37	90%	5%	4%	1%	0%
African Advanced Vaccinology Course	South Africa	AFR	2016	R	E	2	115	58	90%	0%	10%	0%	0%
Masters in Vaccinology	South Africa	AFR	2019	G	E	42	0	0	NR	NR	NR	NR	NR
Advanced Vaccinology Course	France	EUR	2000	G	E	2	1140	60	17%	51%	25%	2%	5%

*Notations: Regions:* AMR: Americas, AFR: Africa, EUR: Europe, WPR: Western Pacific, SEAR: South East Asia; *Reach:* N: National, R: Regional, G: Global; *Language:* C: Chinese, E: English, F: French, S: Spanish; NR: Not reported.

**Fig. 1:**
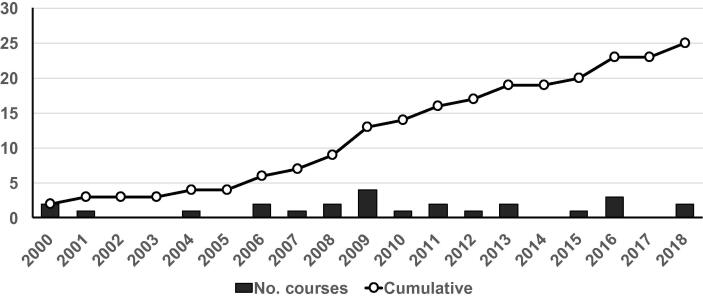
Trend on new vaccinology courses around the world (number per year and cumulative) 2000–2018.

**Fig. 2:**
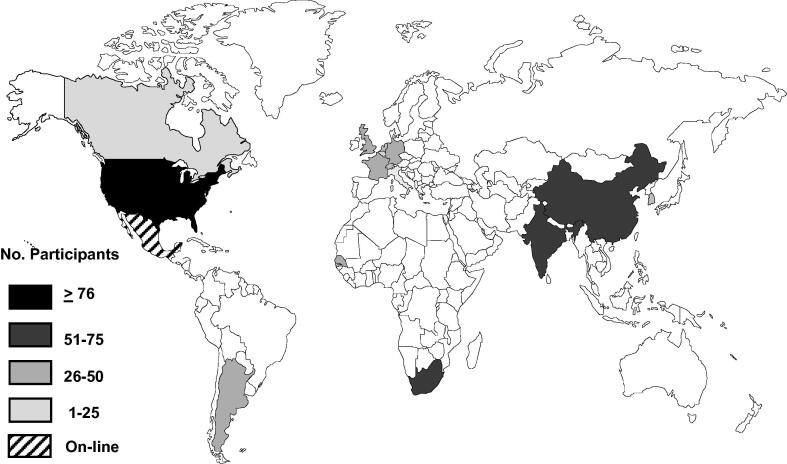
Geographic distribution of advanced vaccinology courses as of 2018.

Since the delivery of first advanced vaccinology course in 2000, 8 additional courses were established by 2008. During the next decade, from 2009 to 2018, the number of courses increased threefold (Fig. 1). The advanced vaccinology courses have also expanded geographically. While the first few courses were located in Europe (France and Germany) and in the Western Pacific Region (South Korea), courses are now available in the Americas (North and South), Europe, South East Asia (India) and the Western Pacific region (China). There are no courses located or serving specifically to Eastern Europe, the Eastern Mediterranean Region and the rest of the Western Pacific Region (Table 1). Courses with a more regional and global reach tend to have larger student participation, except for the country-specific National Foundation for Infectious Diseases (NFID) course in the United States (Fig. 2).

**Fig. 3:**
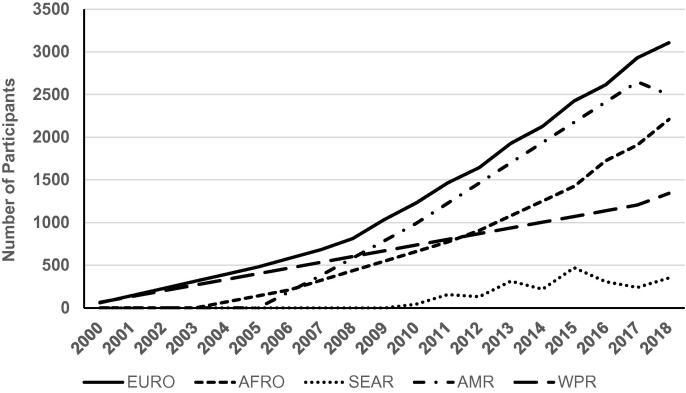
Cumulative average number of participants per year and region to in-person vaccinology courses 2018.

In line with the significant increase in the number of courses, the number of professionals who have been trained overall also has dramatically increased. Courses located in the European and the Americas regions have had the largest number of trainees (n = 3106 and n = 2496 respectively) followed by those in the African region (n = 2209); while those located in Western Pacific (n = 1343) and South East Asia regions trained the least number of participants (n = 352) (Fig. 3). On-line only advanced vaccinology courses have been established in Mexico, France, and Switzerland, and are estimated to have had an additional 9800 participants who took these web-based trainings.

### 3.1 Participants’ selection

Most courses (n = 21, 81%) have a selection process for its participants with exceptions being two of the on-line courses in Mexico and France, and three courses in the Americas that are dedicated to immunization managers and public health officials and do not restrict the number of participants. Of those selecting their participants, they have a variety of educational pre-requisites to attend the course, that at a minimum includes having a professional degree. The majority of courses select participants who are working in public health (mean: 34%, range 33–90), or academia (mean: 33%, range 20–90), with fewer including participants from vaccine industry (mean: 18%, range 10–80) and only few attending to regulators (mean: 5%, range 0–20). While most participants are mid-career level, the entire career range is seen in many of these courses.

### 3.2 Course structure and evaluation

All courses use an educational syllabus that includes vaccine immunology, epidemiology, clinical trial design, immunization program strengthening, vaccine safety, ethics and topics on novel vaccines (malaria, dengue, cholera, Ebola, etc.). All courses deliver most of this content via interactive lectures by international or local experts in vaccinology. Most (n = 20, 77%) of the courses include small group exercises, case studies or special workshops as part of the teaching activities. The majority of courses are delivered in English (n = 20, 77%), with few courses (n = 2) providing simultaneous translation, while others are being delivered in French, Spanish or Chinese.

While the majority of courses (n = 20, 77%) have a course evaluation system in place, most depend on direct observations by the coordinator and paper-based forms completed by participants. Only 30% of those with a formal evaluation system are using an electronic-based platform for their assessment of the course that allows them to provide real-time feedback.

### 3.3 Post-course activities

Almost half (N = 12, 46.1%) of the courses reported having formal post-course activities. These included 7 (27%) with web-based lectures, 3 (12%) with alumni newsletters, one (4%) with periodic alumni meetings, and 3 (12%) with other various activities. Nine (35%) of the courses reported having an active alumni network with periodic or regular contact with previous participants. In addition, some of the alumni from these courses have been the catalytic for the development of new courses in different regions in the world, bringing vaccinology education closer to the areas of most need, creating a cascade training that has expanded the work force in vaccinology.

### 3.4 Outcomes and impact

Two programs have attempted assessments of the long-term impact on participants’ careers, yet no program systematically collects this information to determine the short, medium- and long-term outcomes. The Siena Masters in Vaccinology is currently finalizing an 11-year analysis of the impact of the course over the years (Costa Clemens SA. personal communication). Nevertheless, informal communications and anecdotal observations suggest that many vaccinology course alumni are now part of global, regional and national immunization decision-making committees, hold strategic posts in governmental and non-governmental organizations involved with immunizations, or have successful careers in the vaccine industry or in academia.

### 3.5 Ongoing and future challenges

Vaccinology course directors were asked what were their most recurrent and pressing challenge that their vaccinology course was facing. The issue of sustainability of funding for the course was reported by 11 (42.3%) of the 26 courses, while funding for participants, especially from low and middle-income countries was emphasized by four (15.4%). Except for courses in the Americas, more than half of the courses were challenged by funding forecast. The second most important challenge was the recruitment and time of expert faculty (5, 19.2%) which affected mainly the courses in Europe, the Americas and the Western Pacific Regions (Table 2). Course directors were also challenged by participants’ recruitment, the variable educational and experience background of participants, the logistic to organize the courses especially in Africa including participants’ accommodations and distance, and the struggle to update the courses’ content given other commitments of faculty and coordinators. For one of the online courses, the major challenges were the syllabus content update and the dropout rate of participants.

**Table 2:** Top challenges expressed by Vaccinology Courses around the world by region, 2018.

Challenge	WHO Region					Total (n = 26)^1^
	AFR (n = 6)	AMR (n = 4)	EUR (n = 11)	SEAR (n = 2)	WPR (n = 2)	
Funding forecast^2^	6 (100%)	0	7 (64%)	1 (50%)	1 (50%)	15 (58%)
Recruitment and time of lecturers	0	1 (25%)	3 (27%)	0	1 (50%)	5 (19%)
Recruitment of participants	0	0	1 (9%)	0	0	1 (4%)
Diversity of participant’s education	0	2 (50%)	0	0	0	2 (8%)
Distance for organization^3^	1(17%)	0	0	0	0	1 (4%)
Accommodations for participants	0	0	1 (9%)	0	0	1 (4%)

1 Sum of the columns is 25 as 1 course did not answer the question on challenges, so total percent are calculated from all courses (n = 26) responding the survey.

2 Funding forecast: income needed to sustain the course (travel, venue, lodging, etc.).

3 Distance for organization: Some courses are organized by Institutions away from the venue, so this refers to the remote coordination and logistics that organizers have to secure from abroad.

## 4 Discussion

Since 2000, advanced vaccinology courses have proliferated to meet the demand of formal vaccine science education in almost all WHO regions of the world with the exception of Eastern Mediterranean Region, the Eastern area of Europe and much of the Western Pacific Region. These courses have contributed to the growth in the development of the immunization workforce across many disciplines. No formal impact analysis of many of courses has been attempted yet, with the exception of an initial survey done in 2010 to the ADVAC alumni and an ongoing impact assessment by the Siena Masters in Vaccinology. However, many alumni anecdotally have gone on to grow in their vaccinology responsibilities and contributions and have taken positions of vaccinology leadership at regional and global public health institutions and industry. Without a systematic impact analysis, is not clear what aspects of these courses have proven to be most useful and impactful.

Most alumni from the advanced vaccinology courses, are professionals in their mid-career, therefore, it is not surprising that short intense courses are the most frequent educational format used to deliver vaccinology training. As vaccinology training becomes more popular and expands to farther to reach areas, new educational formats are being created, from on-line mass educational platforms to more longitudinal master-in-vaccinology programs [8].

The Bill and Melinda Gates Foundation, along with many of the host academic institutions and other national foundations and governmental organizations have been at the forefront of providing the funding for this upsurge in advanced vaccinology training of the workforce. Despite their great contribution, funding sustainability is perceived as one of the major threats by most vaccinology course directors.

There are limitations to this landscape analysis. Our data were not exhaustive and represented a subset of the 39 courses initially identified. There were likely additional existing courses meeting the inclusion criteria although meeting attendees did not identify any at the time of the workshop e.g.; a course in Italy targeting a national audience and which did not have any information posted on the web and that was identified later as part of the follow-up of the global workshop and establishing a vaccinology e-Portal (www.global-vaccinology-training.com). In addition, the situation changes overtime in view of the challenging sustainability of some of the courses. Still the results are probably giving a fairly good landscape of the situation globally. As noted there has been no systematic assessment of the impact of these courses, but rather circumstantial evidence that requires a future systematic assessment. Research into the impact, both on career path and on daily work of different types of participants and overall would be helpful to further develop and optimize the courses. Similarly, although some courses have alumni networks, it is not clear how helpful these are, and if more is needed to support them as in-depth details were not part of the survey. Furthermore, these has not been a systematic assessment to see who is missing i.e. gaps in participants recruited beyond those noted.

This landscape analysis highlights the similarities and differences in what, where, when, how and for whom these courses are offered. Further tracking of key indicators and future review of their content and breath of training will be relevant, as well as a reassessment of the range of participants trained to see if gaps has been rectified.

The global vaccinology training website that has been mentioned above will help identify further additional vaccinology courses that will want to be listed on the electronic registry. A primary aim of the platform is to facilitate identification of suitable training by those looking for such courses. This platform will also help strengthen a workforce network to advance vaccinology around the globe.

## Declaration of Competing Interest

The authors declare that they have no known competing financial interests or personal relationships that could have appeared to influence the work reported in this paper.

## Annex A . Current members of the global vaccinology training collaborative^f^

Rachel AFAAYO, East Africa Centre for Vaccines and Immunization (ECAVI), Uganda;

Lizzelott ANDERSSON, Carlos Slim Foundation, with endorsement of the National Autonomous University of Mexico, Mexico;

Sandra ANGELE, Fondation Mérieux, France;

Narendra ARORA, The INCLEN Trust International/Child Foundation, India;

Rebecca ASHFIELD, University of Oxford/Jenner Institute, UK;

Edwin ASTURIAS, University of Colorado, USA;

Amanda SHORTELL BEAL, Bill & Melinda Gates Foundation, USA

Anuradha BOSE, Christian Medical College, India;

Béhazine COMBADIERE, INSERM, France;

Sue Ann COSTA CLEMENS, Institute for Global Health-University of Siena, Italy;

Clare CUTLAND, University of the Witwatersrand, South Africa;

Carine DOCHEZ, University of Antwerp, Belgium;

Philippe DUCLOS, University of Geneva, Switzerland;

Amy FINAN, Sabin Vaccine Institute, USA;

Adam FINN, University of Bristol, UK;

Veronica GAMBILLARA FONCK, Limmatech Biologics, Switzerland;

Benjamin KAGINA, University of Cape Town, South Africa;

Ndeye Coumba Touré KANE, Université de Dakar, Sénégal;

Jean-Louis KOECK, Direction Centrale du Service de Santé des Armées, France;

Jean-Pierre KRAEHENBUHL, Health Sciences E-training Foundation, Switzerland;

Paul-Henri LAMBERT, University of Geneva, Switzerland;

Ann LINDSTRAND, World Health Organization, Switzerland;

Julia Anne LYNCH, International Vaccine Institute, Korea;

Noni E MACDONALD, Dalhousie University, Canada;

Oliver Ombeva MALANDE, East Africa Centre For Vaccines and Immunization (ECAVI), Kenya;

Lindsay MARTINEZ, Freelance Consultant, Switzerland;

Claude MERIC, Freelance Consultant, Switzerland;

Liudmila MOSINA, WHO Regional Office for Europe, Denmark;

Rudzani MULOIWA, University of Cape Town, South Africa;

Hanna NOHYNEK, Finnish Institute for Health and Welfare, Finland;

Bénédicte PANSIER, Fondation Mérieux, France;

Jade PATTYN, University of Antwerp, Belgium;

Stéphane PAUL, Université Jean Monet, France;

Armelle PHALIPON, Institut Pasteur, France;

Audino PODDA, GSK Vaccines Institute for Global Health, Italy;

Lance RODEWALD, Chinese Center for Disease Control and Prevention, China;

J. Anthony SCOTT, London School of Hygiene and Tropical Medicine, UK;

H. Keipp TALBOT, Vanderbilt University Medical Center, USA;

Suresh TIKOO, University of Saskatchewan, Canada;

Jane TIPPING, Faculty of Medicine, University of Toronto, Canada;

Pierre VAN DAMME, University of Antwerp, Vaccine and Infectious Diseases Institute, Belgium;

Likui WANG, University of Chinese Academy of Sciences, China;

Ine WOUTERS, University of Antwerp, Belgium.

## Annex B Courses participating in the global vaccinology training collaborative

Advanced Course of Vaccinology (ADVAC), Mérieux Foundation and the University of Geneva;

Advanced Vaccinology Course in India (INDVAC), Christian Medical College, Vellore;

African Leadership in Vaccinology Expertise (ALIVE) MSc in Vaccinology, The University of the Witwatersrand, Johannesburg;

Afro-ADVAC-African Advanced Vaccinology course, African Leadership in Vaccinology Expertise (ALIVE), The University of the Witwatersrand, Johannesburg;

Annual African Vaccinology Course (AAVC), University of Cape Town;

Chinese Vaccinology Course (CNVAC), Medical School, University of Chinese Academy of Sciences;

Ciro de Quadros Vaccinology Course for Immunization Managers in Latin America, Sabin Vaccine Institute;

Clinical Vaccinology Course, National Foundation for Infectious Diseases (NFID);

Cours international francophone de vaccinologie (CIFV), Université de Bordeaux - Service de santé des armées - Groupe d’études en préventologie;

Diplôme International Inter Universitaire de Vaccinologie (DIUI), University of Dakar;

Summer course on vaccinology for students, Antwerp University;

Vaccinology Course for Health Professionals, East Africa Centre for Vaccines and Immunization (ECAVI);

Epidemiological Evaluation of Vaccines: Efficacy, Safety and Policy, London School of Hygiene & Tropical Medicine;

Indian Advanced Vaccinology Course and Essential Vaccinology Course organized by the Child Health Foundation and the International Clinical Epidemiology Network (INCLEN);

International Vaccinology Course, Institut Pasteur Paris;

International Vaccinology Course, International Vaccine Institute, Seoul;

Latin American Online Vaccinology Course (DILVAC), Carlos Slim Foundation;

Master of Advanced Studies of Vaccinology (IMVACC), University of Lausanne;

Master in Vaccinology and Pharmaceutical Clinical Development, University of Siena, Institute for Global Health;

MOOCS vaccinology, Institut Pasteur, Paris;

Regional Vaccinology Course for African countries, WHO AFRO and The Network for Education and Support in Immunisation (NESI);

Vaccinology in Africa MSc Level Course, Jenner Institute, University of Oxford;

TropEd Advanced Vaccinology Course, Institute of Tropical Medicine and International Health and Charité University, Berlin;

University of Oxford Vaccinology programme (Human and Veterinary Vaccinology, Clinical Development and Biomanufacturing), Jenner Institute and University of Oxford;

Vaccinology and Immunotherapeutics, University of Saskatchewan;

Vaccinology Summer School (vaccines for infectious diseases and cancer), Leiden University Medical School.
